# Continuous quality improvement for prehospital STEMI improved triage rates and achievement of gold standard < 90-min EMS-to-balloon time

**DOI:** 10.1186/s12245-025-00863-x

**Published:** 2025-03-12

**Authors:** Aarish Reddy, Latha Ganti, Anjali Banerjee, Paul Banerjee

**Affiliations:** 1 Innovation High School, Orlando, FL USA; 2 Polk County Fire Rescue, Bartow, FL USA; 3 Orlando College of Osteopathic Medicine, Winter Garden, FL USA; 4 The Warren Alpert Medical School of Brown University, Providence, RI USA; 5 University of Georgia, Athens, GA USA

**Keywords:** ST-segment elevation MI (STEMI), Polk County Fire Rescue (PCFR), American Heart Association Mission Lifeline STEMI

## Abstract

**Background:**

ST-elevation myocardial infarction (STEMI) is a type of myocardial infarction caused by a buildup of plaque or clot in the coronary arteries. There are approximately 750,000 STEMI cases each year in the United States. The American Heart Association's Mission Lifeline initiative aimed to optimize prehospital emergency medical services (EMS) and enhance STEMI triage rates through improved standardized protocol. This study evaluates the implementation of Mission Lifeline techniques by Polk County Fire Rescue (PCFR) on improved EMS-to-balloon (E2B) times and triage rates.

**Methods:**

Data from PCFR, Florida’s 4th largest EMS system, were analyzed quarterly from 2015 to 2023. The study included patients with chest pain that were > 35 years of age.

**Results:**

Among 2,585 patients, the percentage meeting the 90-min EMS-to-Balloon time increased from 74% in 2015 to 84% by the year 2019. The average annual under-triage rate for STEMI decreased from ranging from 2% in 2015 to 4% in 2017 to below 1% after the year 2020, reaching as low as 0% in 2021 and 2023. Over-triage rates initially fluctuated, increasing to a height of 12% by 2017, but decreased to < 3% by 2022 and further dropped to 0.6% of cases were over-triage by 2024.

**Conclusion:**

Implementation of Mission Lifeline procedures, including obtaining pre-hospital 12-lead ECG and hospital pre-activation, significantly enhanced STEMI care. These interventions led to improved E2B times and more accurate prehospital STEMI identification, underscoring the importance of coordinated, protocol-driven prehospital STEMI care in improving patient outcomes.

## Introduction

Within the United States, approximately 750,000 ST-elevation myocardial infarctions (STEMI) cases occur annually [1]. In STEMI, a 30-min delay in reperfusion affects 1-year mortality by 7.5% for 30 min delay; therefore, prioritizing timely triage is crucial [1–3]. Consensus guidelines came to an agreement on a < 90 min time frame for reperfusion for STEMI patients for treatment via percutaneous coronary intervention (PCI) angioplasty [2, 4, 5]. The American Heart Association (AHA) developed the Mission Lifeline initiative in 2007 to optimize prehospital emergency medical care while enhancing triage rates through efficient prehospital EMS rapid calls of STEMI. The role of prehospital EMS in the care system for these patients is crucial and drives the course for patient outcomes. The AHA’s Mission Lifeline protocol implemented by Polk County Fire Rescue (PCFR) has shown continuous improvements in the arena of prehospital EMS alongside a decrease in EMS-to-balloon (E2B) times for primary angioplasty treatment. E2B time specifically refers to two events: the patient entering the emergency room door and the following step within PCI, where a balloon is inflated, allowing a small cavity within the coronary artery for a stent to be implanted to widen the vessel reperfusion of the heart [4]. E2B has strongly decreased from prehospital paramedics providing quality improvement following the AHA Mission Lifeline initiative. In addition to the protocls, PCFR sought feedback from hospital personnel with monthly meetings. The meetings cover prehospital practices, and holding hospitals accountable including bypassing hospitals that lack transparency and fail to meet E2B times. As an intervention for STEMI, PCI is crucial and proven to reduce mortality, morbidity, and hospital stay length by allowing rapid coronary reperfusion and recovery [4]. However, PCI can only be performed within 12 h after the onset of symptoms and, more importantly, within 90 min of entering the emergency room [5]. As such, patients in STEMI need prompt transportation to medical facilities best equipped to achieve E2B times < 90 min from arrival to the medical system [4].

## Methods

Polk County Fire Rescue (PCFR) is the 4th largest EMS system in Florida covering 17 municipalities with 2,010 square miles and over 85,000 patient calls a year [6]. PCFR, a key player in this research, collected data quarterly from January 2015 to December 2023. The PCFR quality and research registry was exempt by the University of Central Institutional Review Board (IRB). PCFR applied Mission Lifeline’s techniques to minimize E2B and develop improved triage rates. PCFR is one of the few EMS systems nationwide that follow the AHA Mission Lifeline initiatives procedure. Inclusion criteria are patients who call PCFR with symptoms of myocardial infarction or report chest pain.

## Results

**Fig. 1:**
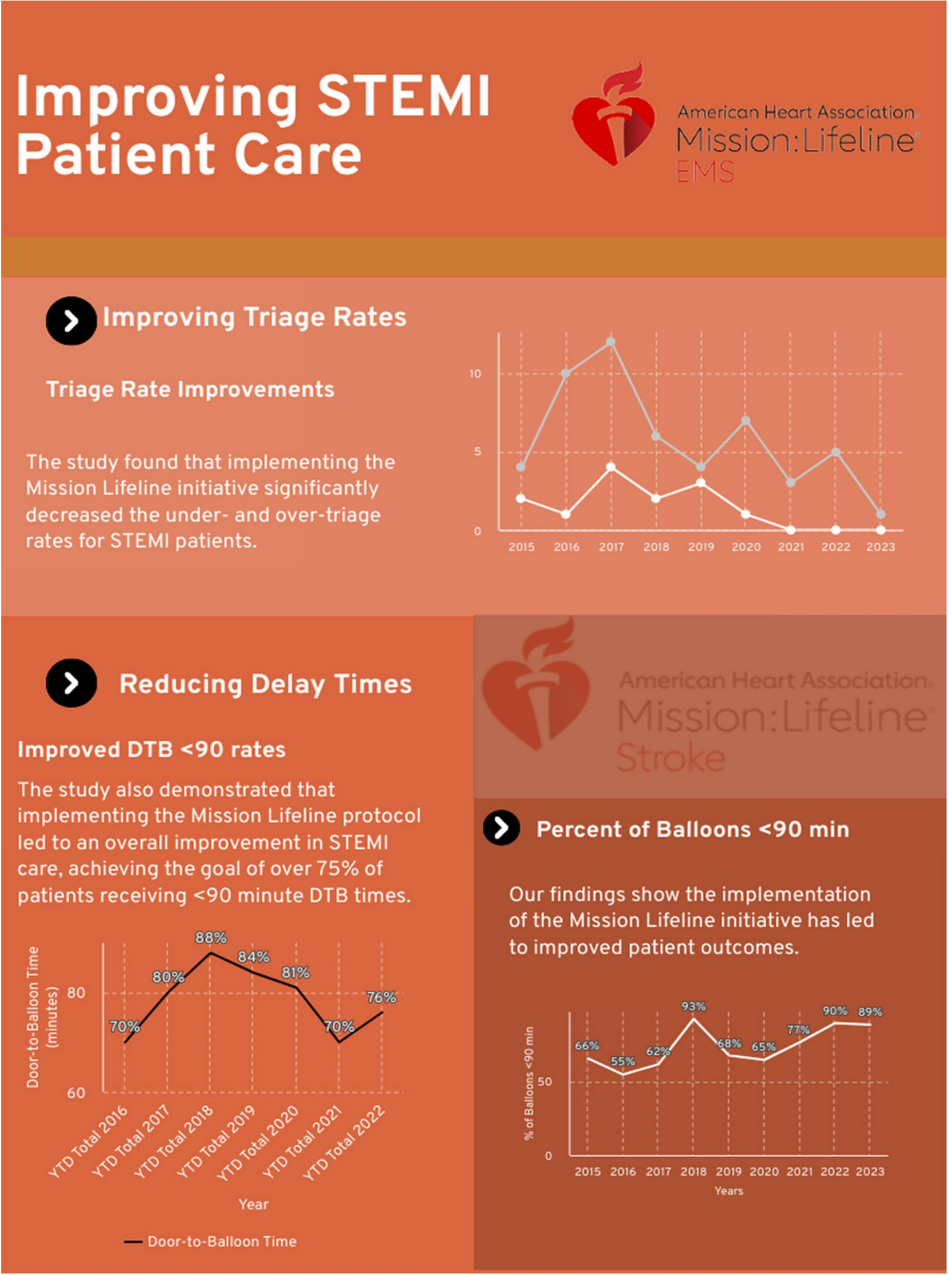
Infographic depicting the results of Mission Lifeline STEMI targets at Polk County Fire Rescue EMS. Note DTB (door to balloon) is used interchangeably with E2B, EMS to balloon time

Among the 2,585 patients transported by PCFR since 2015, when the Mission Lifeline protocol was first implemented, the percentage of patients meeting the E2B time of 90 min steadily increased over the first four years, demonstrating continuous improvement (Fig. 1). By 2015, 74% of patients (*n* = 142) received proper-timing PCI within the E2B window. The response was only the beginning of continuous improvement; by 2019, the rate increased to 84% (*n* = 142).

Despite the challenges posed by the COVID-19 pandemic, the Prehospital STEMI system has been steadily improving. In the years 2019, 2020, and 2021, PCFR presented a notable drop in E2B times < 90 min to 84% in 2019, 81% in 2020, and 70% by 2021, respectively. Narrowly failing to achieve > 75% of patients in less than < 90 E2B times. However, this was accompanied by a significant improvement in triage rates, indicating that the Mission Lifeline tactics successfully combat STEMI through timely triage, even in the face of a pandemic.

**Fig. 2:**
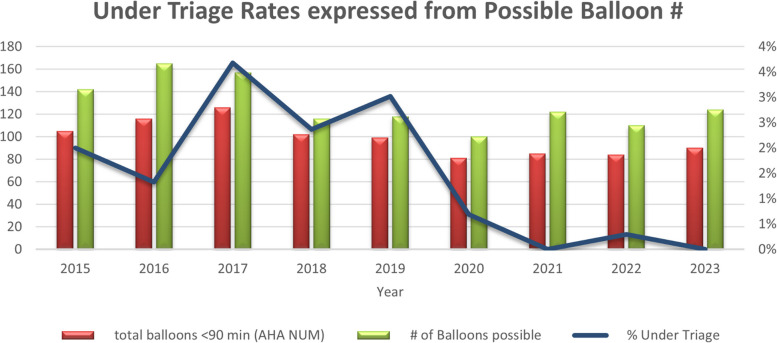
Graph depicts the under-triage rate expressed as a proportion of the total balloon angioplasties performed. The figure illustrates a rapid decline in under-triage rates, comparing the number of patients receiving balloon angioplasty against total balloons deployed within 90 min. Following the implementation of Mission Lifeline, STEMI was more frequently identified in the prehospital stage, contributing to decreased under-triage rates

Under-triage was defined as when the patient had signs and symptoms consistent with a STEMI, but was not considered to have one (“missed”). The average annual under-triage rate for STEMI has shown a consistent downward trend, dropping from 2%- 4% (2015–2017) to below 1% after 2020. This trend continued through the following years, 2021, 2022, and 2023, staying near 0% under triage for STEMI in the prehospital scene, with 2022 showing a rate of 0.3% (Fig. 2). Despite initial fluctuations, the end result of Mission Life at PCFR performed high-quality results in STEMI triage in prehospital care, with only more improvements to come for future STEMI patient care.

**Fig. 3:**
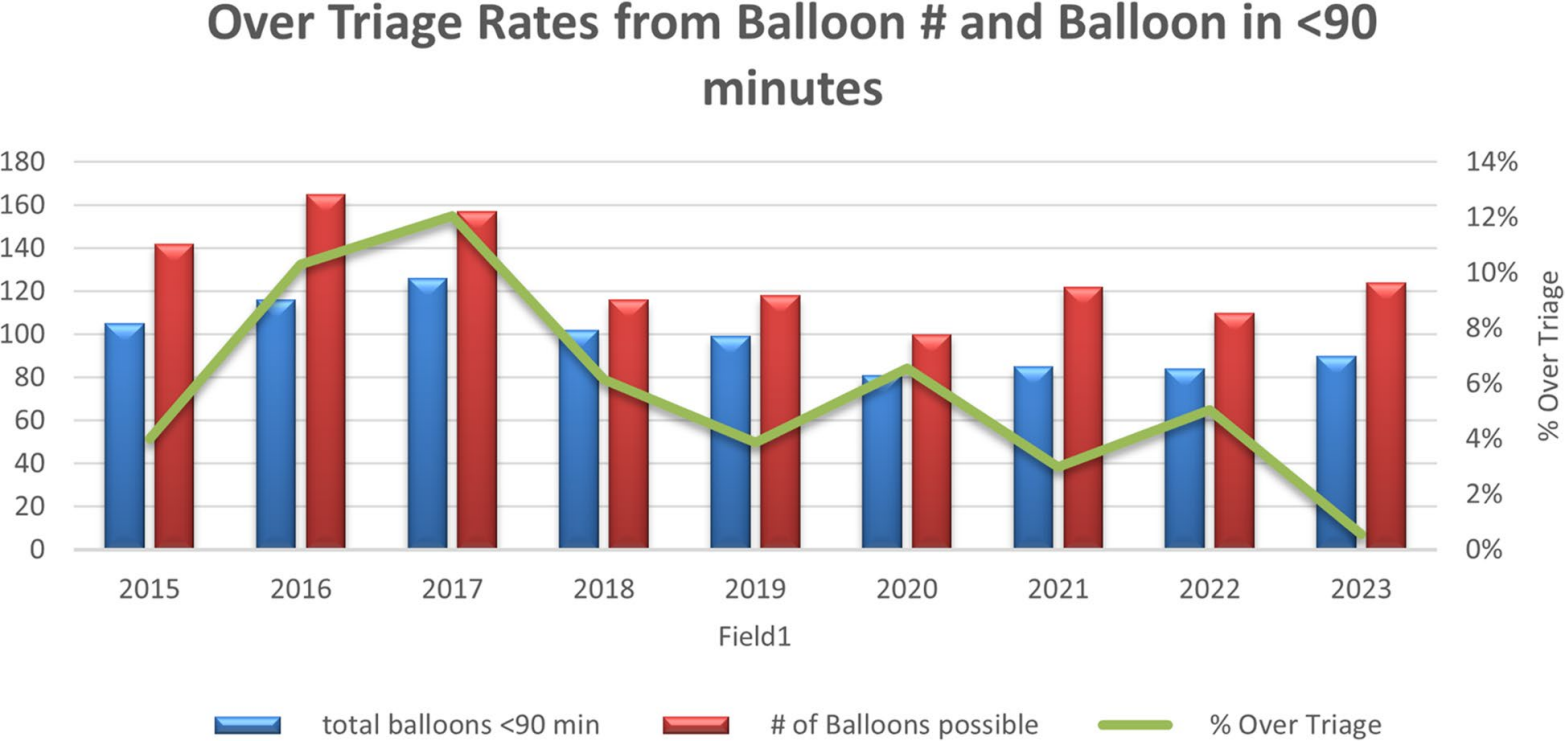
Mission Lifelines initiatives show they perform beyond the correct procedure to create an informed call of STEMI and can avoid false positives. As seen in Fig. 3, the number of false positive calls regarding STEMI decreased. The indicating protocol used both identifies STEMI and avoids invalid identification of STEMI in prehospital settings

Over-triage rates initially fluctuated, increasing from 4% of patients having a false positive call for STEMI at PCFR in 2015. To 10% by the year 2016 and 12% by the year 2017. However, through further practice of the Mission Lifeline protocol, these rates eventually decreased to < 3% by the year 2022 and further dropped to less than 0.6% of cases receiving an over-triage by 2024 (Fig. 3).

The initial protocol, designed to ensure no STEMI was missed, may have led to an overly aggressive approach to triage, particularly when non-STEMI cases presented with symptoms similar to STEMI. Nevertheless, the refined Mission Lifeline protocol and subsequent training gradually reduced over-triage rates over the years, despite the initial increase.

## Discussion

The Mission Lifeline aims to bring quality improvement to the prehospital setting. One method is implementing a 12-lead ECG, which shows benefit in the form of improved under- and over-triage rates. A considerable improvement is the pre-activation and usage of a 12-lead EKG. Found that alerting the hospital to STEMI improves E2B times for PCI to < 90 min compared to STEMI patients arriving without prior hospital alert [7, 8]. EMS pre-STEMI notification to the hospital showed a smaller infarction size and provided improved recovery for 30 days [8]. Less than half of patients with STEMI are treated by fibrinolysis or angioplasty within guideline door-to-balloon times of < 90 min [7–9]. It discovered that 12 lead electrocardiograms improve the management of STEMI, as patients suspected of STEMI often show a sinus rhythm EKG. A continuous second 12 lead ECG has proven effective in a study that showed 8% of patients being identified as having STEMI even when patients previously showed a nonspecific EKG [10].

Continuous quality assurance is an integral process to the success of a prehospital EMS when following the AHA Mission life guidelines. It recommends monthly Question–answer meetings and continuously questions the best hospital for locations based on how to create effective E2B times. Question–answer meetings occur to avoid a significant weakness of STEMI system care: non-PCI-capable hospitals, and the transfer time spent for example [9]. Through communication with hospitals, predictors of delay for STEMI care such as cardiogenic shock, MI, and cardiac arrest can be determined [7–9]. It considers which hospital should transport patients from a prehospital scene. It is in consideration for prioritization to bypass a hospital that fails to meet E2B times or lacks a cardiac catheterization lab.

Mission Lifeline from AHA asserts that the development of transparent hospital feedback should be a factor in formatting the lowest E2B timing. The basis for adapting varying protocol scenarios is that a “Heart alert” that a hospital receives when a STEMI gets called from a prehospital setting speeds up care. When a heart alert in prehospital space is called to an emergency room to pre-activate a catheterization team, the processing of a STEMI E2B timing vastly decreases. E2B time and in-hospital mortality rates are higher when a patient transfer occurs than when a patient arrives at a PCI-ready catheterization lab [11]. Patients face 7% in-hospital mortality compared to 6.7% in PCI-capable-hospital (95% confidence interval (0.8–1.4)) [11]. Kaplan–Meier survival analysis shows that there was a statistically significant decrease in the death rate for those directly admitted to PCI-capable facilities compared with those transferred [12]. Patients transferred from non-PCI-capable hospitals after initial admission faced increased mortality. The mortality rate was 17.4% for direct admission versus 18.7% for those transferred, with statistically significance *p* = 0.017 [12]. Mission Lifeline’s prioritization of PCI-capable hospitals improved patient mortality by removing transfer processes to PCI-capable hospitals or treatment through fibrinolysis.

### COVID-19 pandemic

Beginning in 2019, the coronavirus infection spread, placing a burden on the medical world. While its effect on public health was apparent, cardiovascular disease faced a less noticeable burden. Specifically, in E2B times of STEMI patients, outcomes showed drastically increased median E2B times [13]. Furthermore, a correlation between patients with coronavirus and patients facing elongated E2B times with STEMI (OR 6.31, 95% CI 0.99, 11.63, *P* = 0.02) was prevalent [14]. This statistic means patients with coronavirus or infectious disease face more extended E2B timings. This scenario is likely due to a newfound determinant predictor of delay found for prehospital EMS regarding equipping personnel with protective equipment. Worsened STEMI systems of care are prevalent within our data, providing a notable drop in E2B times to 70% and 76% in 2020 and 2021; however, they are under a continuous ripple of triage.

Furthermore, PCFR door-to-balloon times were falling post-2018, maintaining above 75% of balloons in < 90 yet bleeding into future years, only rising by 2021. Due to ongoing pandemic circumstances, the delay caused by personnel protective equipment is also prevalent from a newfound hesitation to form direct contact with possibly infected or symptomatic patients. The Median E2B times rapidly rose, reaching 290 min from symptom onset to reperfusion [15]. The predicted increase lies in multitudes of factors ranging from fear of hospitalization and receiving coronavirus to delays in prehospital settings from additional infectious disease precautions [14, 15].

## Conclusion

It is crucial to begin in the prehospital stages to maximize E2B times and improve triage ratings. Our findings show that patients with STEMI show the most improved triage rates when following mission lifeline procedures. The pre-activation of an EKG primes hospitals for timely angioplasty, and transparent hospital records provide material for meetings to form best practices regarding STEMI patient treatment.

## Data Availability

No datasets were generated or analysed during the current study.

## Abbreviations

STEMI: ST segment elevation myocardial infarction
PCI: Percutaneous coronary intervention
E2B: Door-to-balloon time
EMS: Emergency medical services
